# High-Grade Serous Ovarian Carcinoma Presenting as Cardiac Tamponade

**DOI:** 10.1016/j.jaccas.2026.108394

**Published:** 2026-05-19

**Authors:** David T. Zhang, Kristiana Hanna, Maoxin Wu, Tahmid Rahman

**Affiliations:** a Division of Cardiology, Department of Medicine, Stony Brook Medicine, Stony Brook, New York, USA; b Department of Pathology, Stony Brook Medicine, Stony Brook, New York, USA

**Keywords:** cardiac tamponade, ovarian carcinoma, pericardial effusion

## Abstract

**Background:**

We present a case report of ovarian carcinoma with pericardial metastasis causing echocardiographic tamponade.

**Case Summary:**

A woman in her 70s with breast cancer gene 1 (BRCA1), B7-H4 positive, high-grade serous ovarian carcinoma with metastasis to the lungs presented with subacute dyspnea. Echocardiogram showed a large, circumferential pericardial effusion of 2.5 cm with tamponade physiology. Emergent pericardiocentesis yielded 420 mL of serosanguinous fluid. Cytology of the pericardial fluid was consistent with metastatic adenocarcinoma of ovarian origin. The patient pursued hospice care, where she later died.

**Discussion:**

Ovarian carcinomas rarely metastasize above the diaphragm or to the heart. Few cases have been reported on cytology-positive high-grade serous ovarian carcinoma from pericardial fluid of a patient presenting with tamponade.

**Take-Home Message:**

A pericardial effusion is the most common manifestation of ovarian cancer metastatic to the heart.

## Visual Summary

**Figure:**
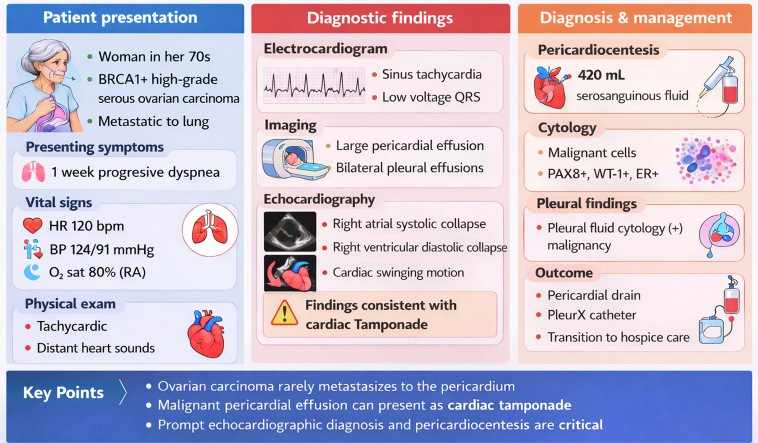

## History of Presentation

A patient in her 70s with breast cancer gene 1 (BRCA1), B7-H4 positive, high-grade serous ovarian carcinoma with metastasis to the lungs presented to the emergency room for 1 week of progressive dyspnea that acutely worsened overnight. On presentation, vital signs were significant for heart rate of 120 beats/min, blood pressure of 124/91 mm Hg, and peripheral oxygen saturation of 80%, requiring 2L of nasal cannula. Physical examination was notable for regular, tachycardic heart rate with distant heart sounds on auscultation.

## Past Medical History

Her oncologic history was significant for high-grade serous ovarian carcinoma metastatic to the lungs first seen on computed tomography (CT) 2 years before presentation. After tissue confirmation, within a month, she received carboplatin, paclitaxel, and bevacizumab followed by olaparib and bevacizumab maintenance therapy. Five months after diagnosis, she underwent a total abdominal hysterectomy, bilateral salpingo-oophorectomy, and omentectomy and later had interval debulking surgery including low anterior resection with primary anastomosis. Two years after diagnosis, given progression of disease, her chemotherapy was switched to carboplatin and liposomal doxorubicin (cumulative dose 96 mg at time of presentation).

## Differential Diagnosis

In a patient with advanced malignancy presenting with dyspnea and a large pericardial effusion, the differential diagnosis includes both malignant and nonmalignant etiologies. Malignant pericardial effusion is a leading consideration, particularly in patients with known metastatic disease, and may occur through direct tumor invasion, lymphatic spread, or hematogenous dissemination. Other malignancy-related causes include treatment-associated pericardial disease, such as chemotherapy-induced or radiation-induced pericarditis. Nonmalignant etiologies also should be considered, including infectious pericarditis (viral, bacterial, or tuberculous), autoimmune disease, uremic pericarditis, and hypothyroidism. In addition, systemic causes such as decompensated heart failure can contribute to serous effusions involving the pleural and pericardial spaces.

## Investigations

**Figure 1:**
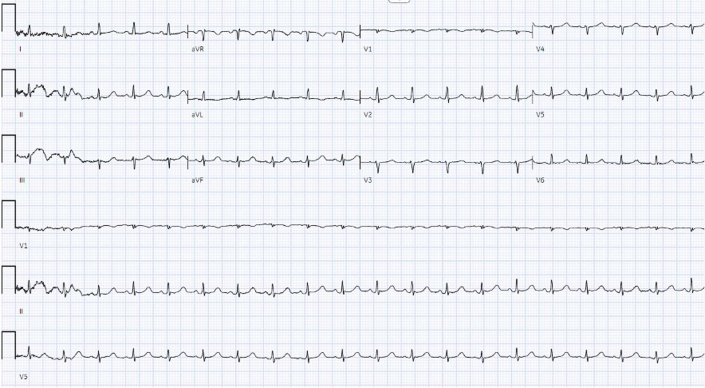
Presenting Electrocardiogram With Sinus Tachycardia and Low Voltage

**Figure 2:**
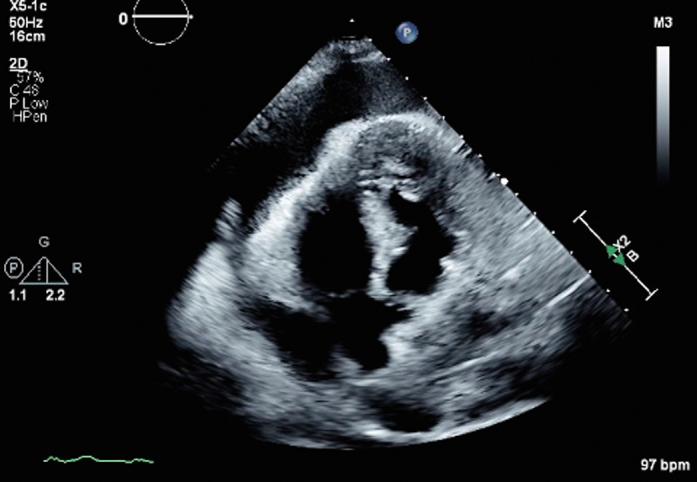
Apical 4-Chamber View Revealing Large Pericardial Effusion and Systolic Right Atrial Collapse

Electrocardiogram showed sinus tachycardia with low voltage amplitude (Figure 1). Electrolytes were unremarkable, thyroid-stimulating hormone was within normal limits, serial high-sensitivity troponins were negative, and N-terminal pro–B-type natriuretic peptide was 389 pg/mL (normal ≤300 pg/mL). Chest x-ray showed pulmonary edema and bilateral pleural effusions. CT pulmonary angiography showed a large pericardial effusion, large bilateral pleural effusions, and small volume ascites. The echocardiogram showed normal left ventricular systolic function with an ejection fraction of 61%, as well as a large circumferential pericardial effusion measuring 2.5 cm in its largest dimension, diastolic collapse of the right ventricular free wall, right atrial systolic collapse, and cardiac “swinging” motion of the heart—findings consistent with cardiac tamponade (Figure 2, Video 1). There was no significant respiratory variation of mitral or tricuspid valve inflow velocities, and the inferior vena cava was normal in size with normal respiratory variation. No prior CTs or echocardiograms were available for comparison of the pericardial effusion.

## Management

She was given intravenous fluids and transferred to the cardiac intensive care unit. Emergent pericardiocentesis via subxiphoid approach yielded 420 mL of serosanguinous fluid, and a pericardial drain was left in place. She had bilateral pleural pigtail catheters placed as well for the pleural effusions.

## Outcome and Follow-Up

**Figure 3:**
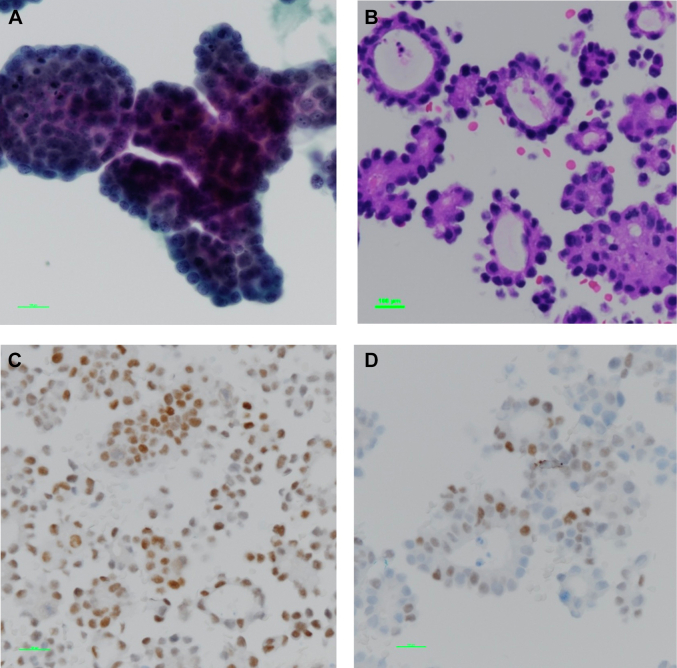
Pericardial Fluid Cytology Confirming Metastatic High-Grade Serous Ovarian Carcinoma Cytopathology photomicrographs: (A) Thin prep slide with Papanicolaou stain shows 3-dimensional clusters of tumor cells. (B) Cell block slide with hematoxylin-eosin stain reveals tumor cells arranged in a flower-like pattern. (C) Immunocytochemical stain with paired box gene 8 (PAX8) demonstrates diffuse positive nuclear stain. (D) Tumor cells are partially positive for estrogen receptor.

**Figure 4:**
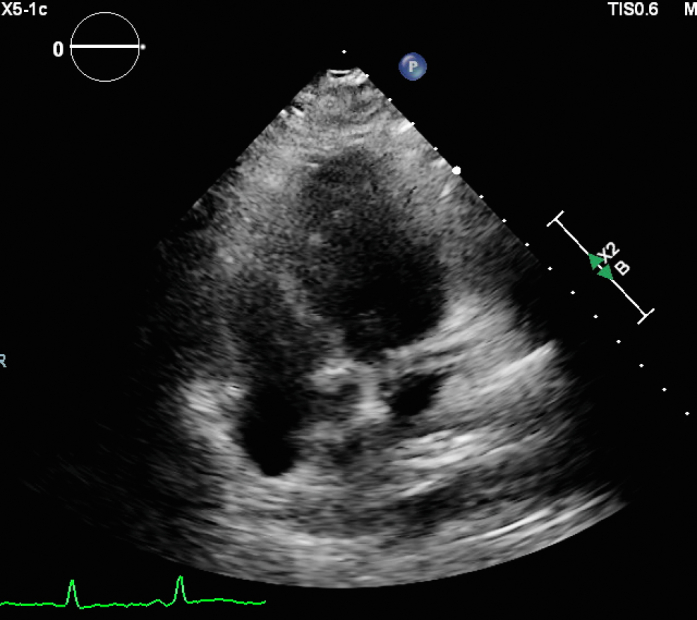
Echocardiogram After Pericardial Drain Removal Demonstrating No Pericardial Effusion

The cytology of the pericardial fluid was consistent with metastatic adenocarcinoma of ovarian origin and tumor cells were positive for paired box gene 8, estrogen receptor, and Wilms tumor 1 but negative for calretinin (Figure 3). Similarly, pleural fluid cytology was positive for metastatic adenocarcinoma of ovarian origin. The pleural thin prep showed tumor cells exhibiting the same cytomorphology as that of the patient's pericardial fluid. Immunostains were attempted including paired box gene 8, estrogen receptor, Wilms tumor 1, and calretinin but were inconclusive because of insufficient cellularity. The patient's pericardial drain was removed on hospital day 11, and the patient's pleural pigtail catheter was replaced with a PleurX Pleural Catheter System (Becton Dickinson). Echocardiogram postpericardial drain removal revealed no pericardial effusion (Figure 4). The patient chose to defer further treatment and was discharged home with hospice.

## Discussion

Ovarian cancer was the third most common gynecological cancer globally in 2020.^1^ Ovarian cancer can be derived from epithelial cells (subtypes include high-grade serous, low-grade serous, endometrioid, clear cell, and mucinous), and the remainder are from other ovarian cell types such as germ cell tumors and sex cord-stromal tumors. Ovarian carcinomas rarely metastasize above the diaphragm and are even more rarely reported to cause pericardial effusion and cardiac tamponade.^2^ The prevalence of ovarian cancer as the primary site of all neoplasms metastatic to the heart is reported to be 10.3% with the most common manifestation being pericardial effusion.^3^

Tamponade is a life-threatening condition that results from pericardial effusion and can indicate disease diagnosis or recurrence.^4^ The amount of pericardial fluid that ultimately leads to tamponade can vary based on the speed of the accumulation. It has been reported that as little as 200 mL can cause symptoms of tamponade if it accumulates quickly, but also as much as 2 L can accumulate with no tamponade if there is slow accumulation.^5^ Clinical tamponade is a life-threatening emergency and should be treated immediately with pericardiocentesis. Pericardial effusions can present indolently but then become clinically significant by an inciting event. It has been documented that even small pericardial effusions can become clinically significant if there are concurrent pleural effusions because of the increased extrinsic pressure.^5^

There have been cases of pericardial effusion from epithelial cell ovarian carcinomas, and there have been fewer cases of tamponade secondary to the pericardial effusion. However, because of the low sensitivity of cytology, there are only 11 cases reported of cytology-positive high-grade serous ovarian carcinoma from pericardial fluid of a patient presenting with tamponade.^6^^,^^7^ Our patient had large bilateral pleural effusions that could have exacerbated the clinical significance of her pericardial effusion.^8^ One case report discusses a patient who had pericardial tamponade following a debulking surgery, possibly triggered by the hemodynamic stress of a debulking surgery in the setting of previously present pleural effusions. The case report discusses the possible utility of pre- or postoperative thoracentesis to help reduce the external pressures of the pleural effusion on the pericardium and therefore decreasing the threshold of tamponade.^2^ This is another consideration for patients like our own who have bilateral pleural effusions and pericardial effusion.

In addition, cytologies for pericardial effusions have low sensitivities, and therefore there are often false negatives.^9^ Therefore, documentation when there is a positive cytology report is imperative in understanding complications of ovarian carcinoma. Of patients who had pericardiocenteses for malignant effusions, those with positive cytologies have been shown to have a poorer prognosis with a median survival of 7.3 vs 29.7 weeks, respectively, compared with those with negative cytologies.^10^ Having more documentation about the prognostication of patients with high-grade serous ovarian carcinoma with cytology-positive pericardial effusion can assist with future implications for management and palliative measures.

**Visual Summary:**
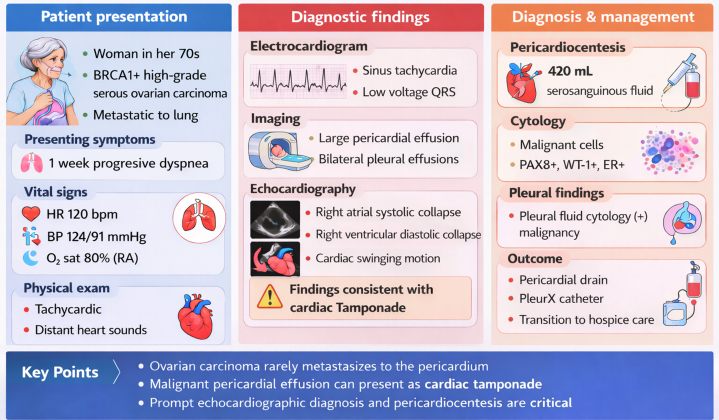
A Woman in Her 70s With BRCA1-Positive High-Grade Serous Ovarian Carcinoma Metastatic to the Lung Presented With Progressive Dyspnea, Tachycardia, Hypoxemia, and Distant Heart Sounds Echocardiography revealed cardiac tamponade. Pericardiocentesis yielded 420 mL of serosanguinous fluid; cytology demonstrated malignant cells positive for PAX8, WT-1, and ER, confirming pericardial metastasis. The case highlights rare pericardial metastasis and the need for rapid recognition and intervention. BP = blood pressure; BRCA1 = breast cancer gene 1; ER = estrogen receptor; HR = heart rate; PAX8 = paired box gene 8; RA = room air; WT-1 = Wilms tumor 1.

## Funding Support and Author Disclosures

The authors have reported that they have no relationships relevant to the contents of this paper to disclose.

**Take-Home Message**

• A pericardial effusion is the most common manifestation of ovarian cancer metastatic to the heart.

## Appendix

Video 1 Echocardiogram demonstrating cardiac tamponade including swinging heart, right atrial systolic collapse, and right ventricular diastolic collapse.
